# Foraging behaviour, habitat use and population size of the desert horned viper in the Negev desert

**DOI:** 10.1098/rsos.220326

**Published:** 2022-06-29

**Authors:** Aziz Subach, Arik Dorfman, Bar Avidov, Adi Domer, Yehonatan Samocha, Inon Scharf

**Affiliations:** School of Zoology, George S. Wise Faculty of Life Sciences, Tel Aviv University, Tel Aviv, Israel

**Keywords:** *Cerastes cerastes*, habitat complexity, habitat selection, inter-sexual dimorphism, movement, predator–prey interactions

## Abstract

The desert horned viper occurs in the dunes of the northwestern Negev desert, Israel. We report on a 2 year study on the viper's behaviour and ecology in its natural habitat. We examined whether the vipers moved faster in a vegetation-dense microhabitat versus an open dune area and detected much slower movement in the former. We nevertheless detected no preference of the vipers for any of the dune areas. We suggest that the vipers trade-off the ease of movement on open areas with prey, which is probably more available in areas with denser vegetation. The activity was higher early in the season and the vipers were mostly active right after sunset, with a second smaller activity peak at sunrise, perhaps searching for burrows to spend the day. Fitting this explanation, movement at the track's end was less directional than at its beginning. We found inter-sexual and between-year differences. For example, females were larger than males in the second year of the study but not in the first one and the population seemed to be smaller in the second year of the study than in its first year. The information we provide on this viper may assist its conservation, as sand dunes are threatened habitats in Israel.

## 1. Introduction

Foraging is one of the most important behaviours because both survival and reproduction require energy. Foraging effort should preferentially be allocated to times and places where its benefit, or the obtained energy, are higher than the costs associated with foraging [1–3]. Such costs are various and include the energetic cost of movement, predation and injury risk, and missed-opportunity cost [4–6]. Each cost can change in interaction with biotic and abiotic factors. For example, moonlight may increase predation risk by making potential prey more conspicuous to their predators, and unsuitable temperature, either too low or too high, may increase movement costs [7,8]. Foraging benefits vary too, and hungry animals benefit more from foraging than satiated ones [9,10]. Habitat structure is an important factor, which can affect the foraging benefits and costs in multiple ways. A complex habitat, or a habitat with extensive surface irregularities [11], may increase foraging costs on the one hand, as it increases travel distance and time [12]. On the other hand, it may also attract more prey, increasing the probability of prey encounter [13,14]. Thus, preference for complex habitats should be probably determined by a trade-off between these two considerations.

Sand dunes often comprise three distinct microhabitats: shifting sand free of vegetation, vegetated semi-stabilized areas and fully vegetated stabilized ones [15]. This distinction is not rigid, and dune areas could be stabilized also owing to soil crust instead of dense vegetation [16]. These microhabitats differ in their complexity level, with shifting sands being usually the least complex and the stabilized dune areas being often the most complex. Animal preference for one of these areas is species-specific, as generalist species usually prefer stabilized areas, and more specialized (psammophile) species occur more often in shifting sands [17]. Such a strict separation is not always the case as other species, either psammophiles or generalists, can be found all over the dune. Preference also depends on the competition with other species and predation risk, and along with their intensification, animals switch to secondary areas [18,19]. Sand dunes in Israel experience a gradual process of stabilization for multiple reasons, including the absence of grazing [20]. Dunes are generally sensitive to external disturbance because their existence depends on the interaction among multiple factors, such as wind, sand movement, vegetation and soil microbial crust [21]. With the process of dune stabilization, the psammophile species are replaced by generalist ones [22].

Psammophile species need, on the one hand, to overcome problems associated with living on sand. For example, movement and digging burrows are more challenging in sand compared to other habitats [23,24]. On the other hand, sandy habitats provide advantages in deserts, such as holding water better, leading to higher productivity and animal activity than adjacent desert areas [25]. Psammophile species have evolved adaptations enabling them to take advantage of the unique features of their habitat. For instance, such species can bury themselves rapidly in the sand and have either long limbs with toe fringes or reduced limbs, both enabling efficient movement on/in the sand [26,27]. Quantifying animal activity on dunes is easy because they leave tracks, like tracks left by animals moving on snow [28,29]. Reading tracks is less expensive, disturbs the animals less than electronic tracking tools and provides more detailed data on behaviour, such as entering a burrow or digging below the sand. It also does not harm the animals.

The communities of desert sand dune rodents have been intensively studied [19,30]. Although the rodents' predators, such as barn owls, vipers and red foxes, affect their foraging behaviour [31,32], such and other similar predators have been less frequently studied. An exception is the sand viper, *Cerastes vipera*: many aspects of its foraging behaviour, such as hunting method, foraging mode and inter-sexual behavioural differences, are known [33–36]. That said, we know almost nothing about another common viper in the Sahara Desert in general and the western Negev in particular—the desert horned viper (*Cerastes cerastes*). Except for information on its venom, taxonomy or distribution [37–39], there are currently no scientific studies on any aspect of its ecology or behaviour in its natural habitat. This viper is not surveyed by the International Union for Conservation of Nature, and it would be of interest also to estimate its population size. Furthermore, this viper shifts between foraging modes, as it either ambushes prey or actively searches for it, similarly to its relative *C. vipera* [33]. Switching between foraging modes may depend on various factors, such as prey abundance, type, size, spatial distribution, movement properties and predator hunger level [40–42].

The goal of this study is to provide behavioural and ecological details on *C. cerastes*, to facilitate future research and potential conservation. We began by conducting an experiment examining whether the viper's movement differs in microhabitats differing in their complexity level, i.e. a microhabitat simulating dense vegetation and an open dune. We then examined whether the viper is more often present in the open dune than in stabilized or semi-stabilized areas. We further explored the factors affecting the viper movement, estimated population size and reported on inter-sexual and between-year differences.

## 2. Material and methods

### 2.1. Habitat description and general methodology

**Figure 1.**
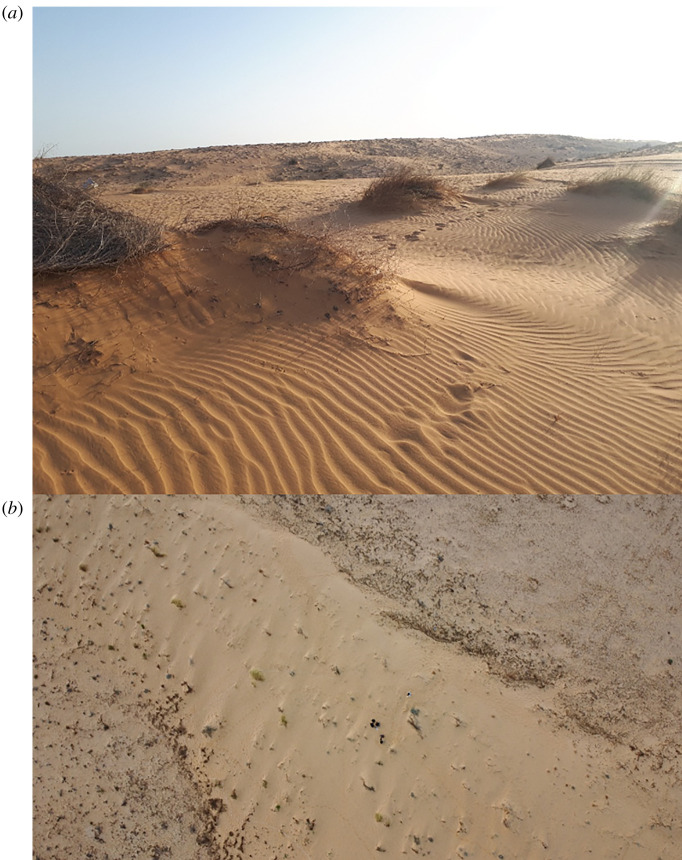
A view of the habitat in the northwestern Negev desert, Israel (*a*) from a human perspective and (*b*) from above (photographed by the authors using a drone). The central area comprises shifting sands whereas the upper-right and lower-left sides are semi-stabilized/stabilized areas.

**Figure 2.**
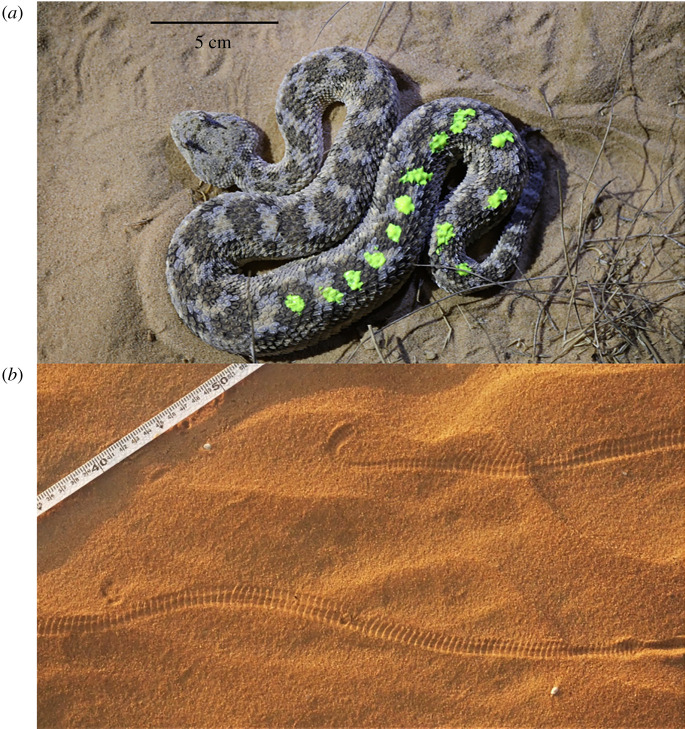
(*a*) The studied viper, *Cerastes cerastes*, which is marked with non-toxic paint. (*b*) Sidewinding tracks of the viper.

The study was conducted on 28 nights (20.00–6.00) in June to September 2020 and April to October 2021 in the sand dunes of the northwestern Negev (Holot Agur; 30.9280 N, 34.4130 E; figure 1). The area is the western extension of the Sahara and Sinai deserts. It comprises mostly shifting sands and semi-stabilized dunes (figure 1). The perennial and annual cover in 1985 was 2.8% and 10.5%, respectively [43]. Annual cover changes among years depending on precipitation in winter. The mean annual rainfall is approximately 100 mm, and the mean January and July temperature is 11 and 26°C respectively [44]. We searched for *C. cerastes* tracks on the sand left by the vipers beginning at sunset until sunrise (figure 2). The viper mostly moves using sidewinding, a common movement method of viperids on sand [29,45]. Once tracks were found, we followed them in both directions to locate the burrows from which the viper started moving to its current location. The trail was marked using poles that were stuck in the sand every several metres and its length was later measured with a measuring tape. Once the viper was detected, it was gently caught with a hook and placed in a large plastic bag. We weighed the viper using a Pesola hanging scale (accuracy of 0.1 g) and determined its sex. Each individual was marked with an identification number painted on the back using a UV glowing colour (figure 2). The paint, which is non-poisonous and approved for the usage of children, holds until molting, which takes place in winter/spring. Therefore, the marking is valid for a single year. We chose this method because it does not harm the viper and can be applied rapidly in the field. Before being released, the viper was photographed next to a ruler for later measurement of its total length (head-to-tail), done using the software ImageJ [46]. The capture location and time were marked using the Google Maps application on a mobile phone. A microclimate station was positioned and recorded the temperature and humidity. No harm was done to any animal in the study. We obtained the required permits from the Israel Nature and Parks Authority.

### 2.2. Maze experiment

**Figure 3.**
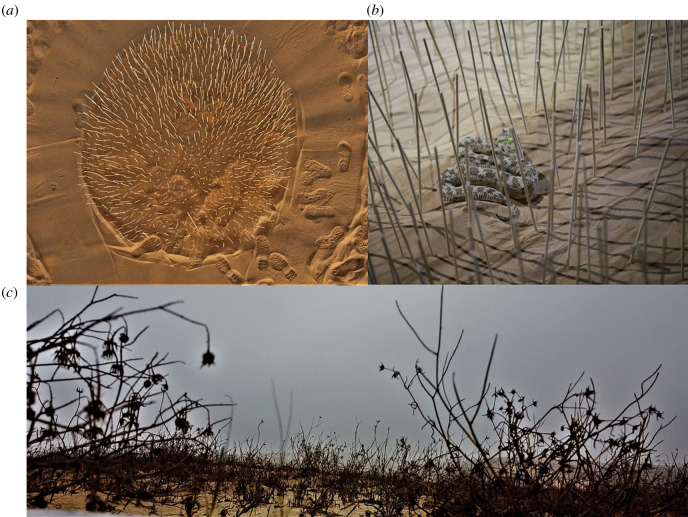
(*a*) The maze was created by deploying 1045 poles in a circle to simulate a densely vegetated area. The viper was placed in the centre. (*b*) The maze from the viper perspective. (*c*) A comparison to a densely vegetated area or the dune stabilized area, which the maze imitates.

To examine whether habitat complexity affected movement speed and thus increased energy expenditure by the viper, we created a maze by placing 1045 poles in a circle in a regular distribution pattern (*d* = 1.2 m; density of 678 poles m^−2^) in a vegetation-free part of the dune. This structure resembles the vegetation in stabilized dunes as seen from the viper's point of view (figure 3), but still allows the viper to move freely inside the maze. A space was left in the maze centre, where the tested viper was released. We watched the viper using a UV flashlight for brief moments. We measured how long it took the viper to leave the maze (598 ± 305 s, [60, 1083]; mean ± 1 s.d., [range]). The viper was then allowed to move freely on the vegetation-free dune for the same time and we marked its movement track with poles. The distances moved inside and outside the maze were measured using a measuring tape based on the tracks left by the viper without disturbing it or approaching it during the experiment. We compared using a paired *t*-test the distance travelled by the viper for the same time. The movement distances were square-root-transformed because they deviated from a normal distribution. This experiment comprised five and eight vipers in 2020 and 2021 (conducted identically), of which all except one were males. Some of the tests were conducted on the same night. In this case, the time interval was at least an hour, during which the sand was swept and cleaned. Statistics were done using Systat v. 13 (Systat Software, Inc., San Jose, CA, USA).

### 2.3. Microhabitat choice

We examined the microhabitat choice in three complementary ways: (i) in 2020, we photographed 15 trails using a DJI Mavic mini drone, each trail in multiple photos. In parallel, we photographed 13 slopes on the dune once more in multiple photos. We chose high points and slopes that include all three dune sections. We examined using a *t*-test whether the plant cover (%) in areas where the viper was active differed from the randomly chosen sites. Plant cover was evaluated by circling the plant area in ImageJ dividing it by the total photo area; (ii) we measured the track of each viper in these 15 trails moved in open areas and semi-stabilized/stabilized areas. We calculated the proportion of movement in open areas out of the total track length and used a one-sample *t*-test to examine whether the proportion differs from the null expectation of 0.5; and (iii) in 2021, we mapped the dune according to stabilized, semi-stabilized, and shifting sand areas using the drone photos and calculated the proportion of each. We calculated how many vipers were spotted in each section of the dune. We compared using a *χ*^2^-test the observed number of captured vipers in each dune section to the one expected by chance considering the relative area of each section. The proportions were arcsine-transformed.

### 2.4. Population size

Some of the vipers were captured more than once. We used the Schnabel method (repeated mark-recapture) to estimate the viper population size separately each year [47,48]. This method fits here because marking and captures occurred several times and not only once. The population estimate is a weighted average measure of Petersen estimates:

$$\hat{N}=\frac{\sum_{t}(C_{t}M_{t})}{\sum_{t}R_{t}},$$

where *C_t_* is the total number of individuals caught in sample *t*, *M_t_* is the number of marked individuals just before sample *t* was taken and *R_t_* is the number of individuals already marked when caught in sample *t*. Such indices are not always accurate, so we also simply present the total number of captures and the proportion of recaptures. We also report on the distances between two successive captures and compare them to the maximal movement distances documented here.

We examined whether the year and month affected the probability to capture one of the two sexes. For this purpose, we used two *χ*^2^-tests with year versus sex and month versus sex. The single viper caught in April was considered as being caught in May. When considering the year of capture, we referred only to vipers of known sex on their first capture (*N* = 23 in each of the 2 years). When considering the month of capture, we referred to first captures and recaptures (total *N* = 75).

### 2.5. Movement

In 2020, the length of the trail left by 37 vipers during the night (20.00–6.00) was measured in the morning using a measuring tape. In 2021, we tracked 13 vipers using a GPS tracking application on a mobile phone. There is a tight similarity between tracks measured with tape and GPS tracking. To verify this, we set 10 zigzag tracks each of 10 m, measured by tape and measured each using GPS tracking. The resulting measurements were 10.28 ± 0.75 (mean ± 1 s.d.), i.e. a deviation of 2.8%. We examined using a mixed linear model whether the body mass, sex, year and month affect the viper movement. The viper identity was referred to as a random variable and movement length was square-root-transformed because it deviated from a normal distribution.

In 2021, we documented whether the viper was captured moving or in ambush position in each of the 40 capture events. We examined whether month and sex were correlated with foraging status using logistic regression. For 11 vipers tracked in 2021, we possess hourly measurements of the distance moved from the time they were spotted until they ceased activity (either entering a burrow or being fully buried in the sand for more than an hour). Such measurements were obtained by following the tracks and placing a pole next to the viper location each hour. We did not constantly follow the vipers in order not to disturb them. We calculated the proportion of vipers active each hour (between 20.00 and 6.00 the next day) and examined using a model selection procedure (Δ Akaike information criterion (AIC)) whether a linear (*y* = *ax* + *b*) or a polynomial (*y* = *ax*^2^ + *bx* + *c*) function described better the link between time and proportion of the vipers active. The proportion of the vipers being active is perhaps a better measure than the movement distance itself, as the distance can be affected by other factors, such as month, and it hence increases the background noise.

For 12 of the tracks, we possess coordinates taken every 0.65–0.97 m (average per track). We calculated the slope between every two successive coordinates and then the slope between two lines using the following formula:

$$\tan(\theta )=\left|\frac{m2-m1}{1+m1m2}\right|$$

where *m*1 and *m*2 are the slopes. Lower and higher values of *θ* indicate more directional and less directional movements, respectively. We asked whether there were differences in the movement directionality between the beginning and the end of the track and whether there was a link between track length and movement directionality. To answer the first question we calculated for each track the mean angle (*θ*) at the first and last 5, 10 and 20 m. We used three paired *t*-tests to compare the mean angles. To answer the second question, we calculated the mean angles over the whole track. We examined a possible link using a Pearson correlation.

### 2.6. Body size

We examined the body mass, length and mass-to-length (residuals of linear regression) of the vipers. We included the viper identity as a random variable when analysing mass and mass-to-length because some of the vipers were caught more than once (see Results). When analysing length individuals were used only once. The explanatory variables were sex, year, month and the year × sex interaction. We also examined the variance between the sexes considering the mass using Leven's test (based on means). Mass was square-root-transformed as it deviated from a normal distribution.

## 3. Results

### 3.1. Maze experiment

**Figure 4.**
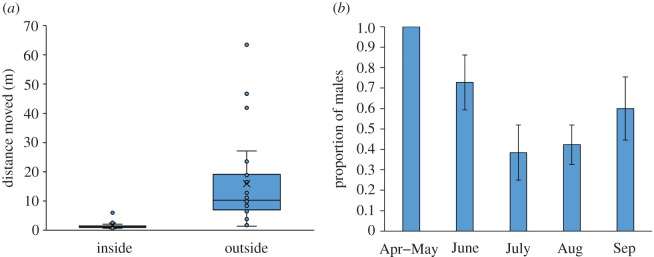
(*a*) The maze experiment results: a boxplot showing that the vipers moved over longer distances outside the maze than inside. (*b*) The proportion of males caught each month (one capture in April was merged with May). Standard errors are calculated according to the formula [*p*(1 − *p*)/*n*]^0.5^, where *p* is the proportion and *n* is the sample size.

The vipers moved more than 10 times longer distances out of the maze area than in it (*t*_12_ = −5.008, *p* < 0.001; figure 4*a*). This result suggests that the vipers are limited in their movement by vegetation and move more rapidly and more easily in the open areas of dunes.

### 3.2. Microhabitat choice

The plant cover in areas in which the vipers moved did not differ from that in random sites (*t*_26_ = 0.771, *p* = 0.447; mean ± 1 s.d. proportion of plant cover: 0.116 ± 0.061). The vipers did not move longer distances in open areas than in semi-stabilized/stabilized areas (*t*_14_ = 0.089, *p* = 0.930). Eight vipers in 2021 were caught in the stable section, 18 in the semi-stable one, and six on shifting sands. The semi-stable area comprised 52.7% of the area, followed by the stable area (34.9%) and by the open area (12.4%). The proportion of vipers spotted in the three dune sections did not differ from the random expectation considering each section area (*χ*^2^_2_ = 0.975, *p* = 0.614). In short, the microhabitat choice analysis did not fit the results of the maze experiment.

### 3.3. Population size

We captured 25 vipers in 2020, of which four were captured twice and four thrice (32% of the vipers were captured again). The total number of captures was 37, of which 12 were recaptures. In 2021, we captured 23 vipers, of which six were captured twice, two were captured thrice, and two were captured four times (47.8% of the vipers were captured again). The total number of captures was 40, of which 17 were recaptures. Using the repeated mark-recapture and the above-mentioned formula, we carefully estimated the population size to be 41 and 33 vipers in 2020 and 2021, respectively.

The distance between pairs of successive captures was 242.4 m ± 202.2 m (four recaptures with known coordinates) in 2020 and 212.9 m ± 138.0 m (14 recaptures) in 2021 (mean ± 1 s.d.; range of both years together: [16, 453.6 m]). Comparing these distances to the daily movement distances of the vipers (up to approximately 790 m per night) made it clear that crossing such distances was possible.

Year had no effect on the sex identity of the vipers caught (*χ*^2^_1_ = 0.807, *n* = 46 captures, *p* = 0.369). However, males were caught more often early in the season in May and June whereas females were more likely to be captured later in July and August (figure 4*b*; *χ*^2^_4_ = 15.108, *n* = 75, *p* = 0.005).

### 3.4. Movement

**Figure 5.**
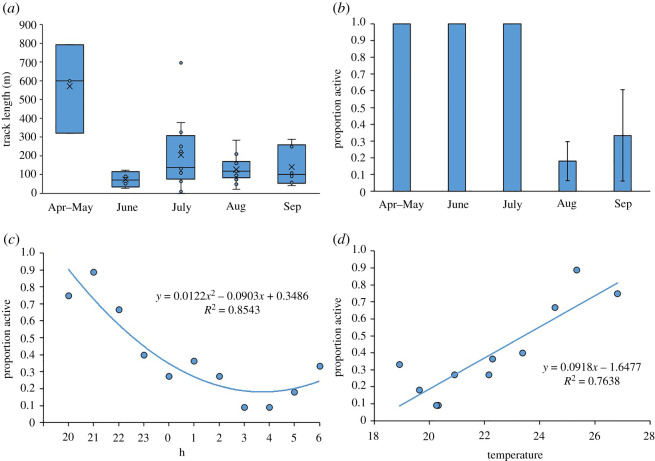
(*a*) A boxplot presenting the track length according to months. One-sample point in April was merged with May. (*b*) The proportion of vipers captured active (versus ambushing) as a function of the month. The proportion of vipers active (*c*) each hour of the night and (*d*) versus the average temperature during each hour of the night.

Month was the only significant factor correlated with movement length (*t* = −3.152, *p* = 0.020) and movement distances were longer in April–May than all other months (figure 5*a*). We could not detect a significant effect of any of the other variables on movement length (sex: *t* = 1.574, *p* = 0.167; year: *t* = −0.661, *p* = 0.533; mass: *t* = 1.192, *p* = 0.278). The vipers moved over 177.0 m ± 172.8 m (mean ± 1 s.d.) per night, with a maximal value of 791.0 m. Regarding foraging status upon capture, there was a strong dichotomy between vipers captured until July, which were always captured while moving, and those captured in August–September, which were more frequently caught while ambushing prey (figure 5*b*; *z* = −2.551, *p* = 0.011). The difference between the sexes was not significant (*z* = 0.231, *p* = 0.817). Hourly inspection of the viper movement indicated that the vipers were more active until 22.00 than later. That said, a polynomial function described the link between time and proportion of the vipers being active rather than a linear function (ΔAIC = 6.79; figure 5*c*). The hour and mean temperature were tightly and negatively correlated (*r* = −0.982; mean temperature at 20.00, 2.00 and 6.00 were 26.8°C, 20.9°C and 18.9°C, respectively). It is therefore not surprising that a strong correlation also exists between mean temperature and the proportion of the vipers active (*r* = 0.857, *p* < 0.001; figure 5*d*).

**Figure 6.**
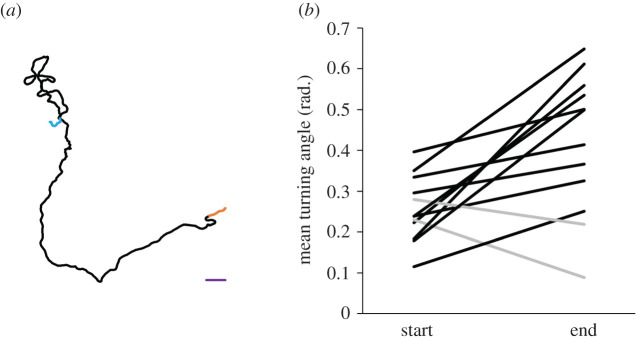
(*a*) An example of a viper track. The track starts at the right side (orange) and ends at the left side (blue), both segments represent 10 m. Scale (10 m) appears as a purple straight line. (*b*) The mean turning angle at the first and last 10 m of the viper movement. Less directional movement at the end is marked with black whereas more directional movement is marked with grey.

Viper movement was more tortuous at the track's end than its beginning (figure 6). The strongest difference was obtained when considering 10 m in each track segment followed by 20 m and 5 m (5 m: *t*_11_ = −2.290, *p* = 0.043; 10 m: *t*_11_ = −3.261, *p* = 0.008; 20 m: *t*_10_ = −2.546, *p* = 0.029). One track was omitted from the calculation when comparing 20 m track segments because the whole track was 22 m. Track length was not linked to the average directionality level (*r* = −0.013, *n* = 12, *p* = 0.967).

### 3.5. Body size

**Figure 7.**
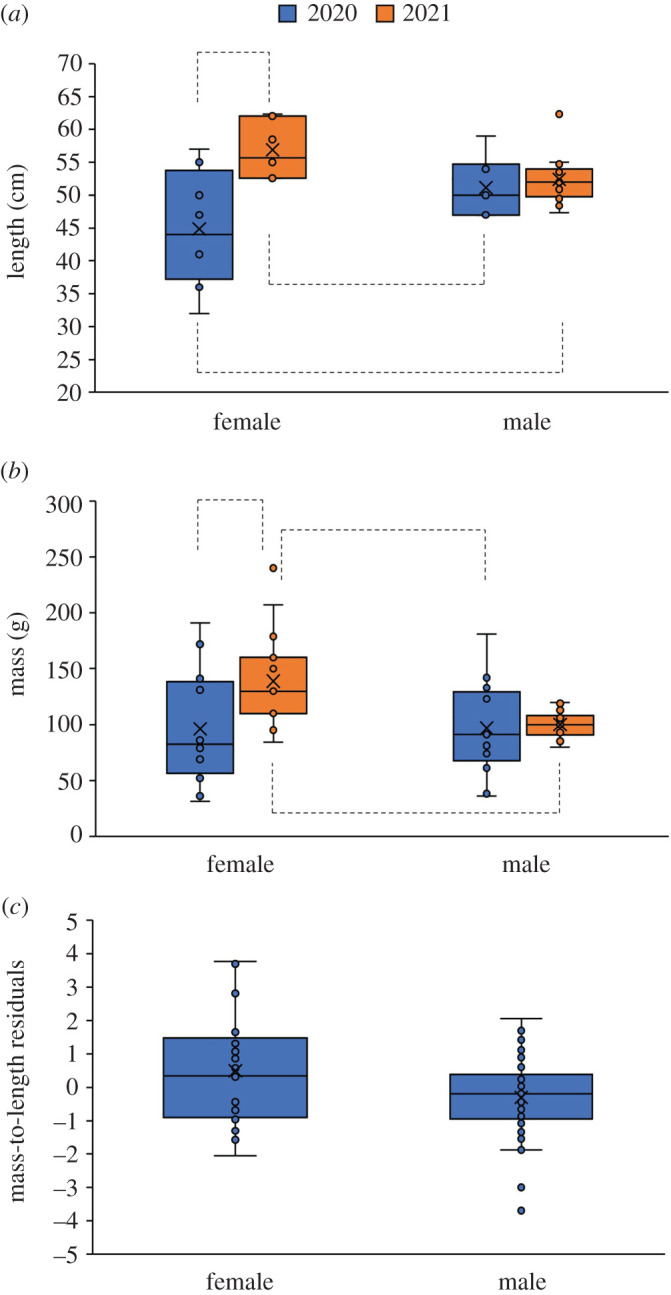
Boxplots presenting the viper's (*a*) length, (*b*) mass and (*c*) mass-to-length ratio (residuals of mass regressed on length) separately presented for each sex. Significant pairwise differences according to post hoc tests are marked with dashed lines.

The vipers were 51.5 ± 6.6 cm length (mean ± 1 s.d.; range: 32.0–62.3 cm). Year and sex interacted to affect the body length: males were of a similar length in 2020 and 2021, whereas females were larger in 2021 than 2020 (figure 7*a*; *F*_1,36_ = 5.838, *p* = 0.021). Year as a main effect was significant (individuals were larger in 2021; *F*_1,36_ = 19.806, *p* < 0.001) but sex was not (*F*_1,36_ = 0.124, *p* = 0.727) as well as month (*F*_1,36_ = 2.411, *p* = 0.129). The vipers weighed 107 ± 39 g (mean ± 1 s.d.; range: 31–240 g). Body mass was affected by an interaction of year and sex (*t* = −2.526, *p* = 0.019). Females in 2021 were larger than males but they had roughly the same mass in 2020 (figure 7*b*). To complete the analysis, month was not significant (*t* = −0.940, *p* = 0.357) as well as year (*t* = 0.066, *p* = 0.948), but sex was (*t* = 2.989, *p* = 0.007). Females had a higher mass-to-length than males (figure 7*c*; *t* = 2.430, *p* = 0.025) with no difference between years (*t* = 0.396, *p* = 0.697) and among months (*t* = −2.064, *p* = 0.053). The year × sex interaction was not significant (*t* = −0.366, *p* = 0.719). Females varied in their body mass almost six times more than males (the variance was unequal: *F*_1,67_ = 9.850, *p* = 0.003).

## 4. Discussion

We present here, to our knowledge, the first behavioural and ecological study of the desert horned viper in its natural habitat and shed light on its habitat use and movement. The conducted experiment demonstrated that the vipers moved much more rapidly in open areas and their movement was obstructed by vegetation. The vipers nevertheless were not found more often in open areas, and we could not detect any preference for any of the dune areas. We suggest that the vegetation-dense areas are richer with prey and burrows that provide hiding places during the day possibly ending in a compromise between food and burrow availability versus the ease of movement. The vipers moved over longer distances earlier in the season than later. They also moved more during the first hours of the night and resumed moving with sunrise although to a lesser extent. The movement pattern was more directional early when movement commenced than later before the track's end. We detected between-year differences as well as inter-sexual differences. For example, more vipers were recaptured in 2021 than in 2020, suggesting that the population in 2021 was somewhat smaller. Moreover, females were larger than males in 2021 but not in 2020.

The maze experiment indicated that movement in an area imitating dense vegetation was much slower than that in an open area, fitting other studies demonstrating elevated movement costs when the habitat gets more complex [12,49,50]. Movement in microhabitats of dense vegetation is probably more costly but not impossible. It could be therefore expected to detect the vipers more often in open areas than both the semi-stabilized and stabilized dune areas. This did not hold true. The areas through which the vipers moved contained the same level of vegetation as other randomly selected areas of the dune. Ease of movement is probably not the only factor the vipers consider when moving and although movement is easier in open areas, prey, such as gerbils, are more available next to bushes in semi-stabilized and stabilized areas [19,51,52]. Updated information on the prey abundance and microhabitat preference is needed to reach more solid conclusions. Furthermore, burrows, required as hiding places during the day, are not available in the open dune area. We made great efforts throughout the study not to disturb the vipers and observe them from a distance. We do not think the vipers were threatened by us as we never observed the viper's stereotypic responses to threats. The vipers probably move over long distances because their habitat is poor with prey. Similarly, other animals possess large home ranges when resources are low [53,54] or reduce movement when satiated [55,56]. Future studies should examine what is the ‘core area’ used by each viper and determine whether the vipers are completely nomad or stick to some areas. Another future experiment could examine not only the viper movement from vegetated to open microhabitats but vice versa and whether the direction of change affects behaviour.

The vipers were most active from sunset until 22.00, ceased activity almost completely at around 22.00 and resumed activity before sunrise (though to a lesser extent). Their activity time during the night may be related to a suitable temperature, i.e. lower than during the day but higher than before sunrise, as temperature sharply decreases after sunset. It could also stem from synchronization with the activity time of potential prey. As seeds are redistributed by wind during the afternoon, it is profitable for granivorous species, such as gerbils, to be active not long afterwards [57], an activity time overlapping with that of the studied viper. As we conducted an observation rather than an experiment, we cannot reach a firm conclusion regarding the dominant factor affecting movement, which should be the theme of a future experiment. The viper movement was less directional at the end of its track than at its start. The reason may be a careful search for a suitable hiding site for the next day. A thorough search of a limited area often involves more tortuous movement, such as animals searching for food, mates or their nest after realizing the target is somewhere nearby [58–60]. More detailed observations of the viper behaviour at this timepoint (the track's end) will be helpful to provide a more accurate interpretation of this change in movement directionality. The only other significant factor affecting movement was month: vipers moved over longer distances early in their active season and employed the ambush strategy more frequently later in the season.

Males were captured more frequently early in the season while females showed the opposite pattern. It could be that males are more active first to search for mates. Later, females are slightly more active perhaps to search for suitable sites to lay eggs or to hunt in order to regain their energy investment in reproduction. This is indeed reported for the closely related *C. cerastes* gasperettii in Saudi Arabia: mating takes place in April–May and females lay eggs until July [61]. A similar pattern of males being more active early in the season or during the breeding season has been demonstrated in other snakes too [34,62–64] but see [65] reporting on males being more active than females and both reaching their activity peak in summer rather than spring. Females were heavier than males but not longer. Females are often larger than males in snakes although the opposite holds true when considering only the family Viperidae, to which the studied animal belongs [66,67]. In the congeneric viper, *C. vipera*, females are larger than males and weigh more for the same body length, as here [33]. Females are probably heavier before reproduction after which they lose considerable weight. The development of eggs and the consequent change in body mass may explain why females vary in body mass more than males. The increase in female size between 2020 and 2021 is perhaps because in 2021 we found no juveniles. This might be a consequence of lower precipitation and more limited resources in the latter year. Data provided from a meteorological station in Kadesh Barnea (approx. 5 km to the south) indicated that 2020 was almost four times rainier than 2021 (105.2 mm versus 27.3 mm; [68]). To reach a firmer link between precipitation and population size, the population size should be followed for several years.

Supporting evidence for the deteriorating conditions in 2021 is the lower estimation of population size on the studied dune (by almost 20%). The population estimation should be taken with caution because the vipers move over long distances, we have no evidence for the absence of either immigration or emigration, and our sampling effort might have somewhat differed among survey events. We nevertheless provide the number of recaptures each year: in 2021, more vipers were recaptured, suggesting a somewhat smaller population. One could expect the vipers to be more active in 2021 than 2020 owing to the drought in 2021 but activity was similar over the 2 years. The reason may be a trade-off between foraging benefits and costs: if the probability to find food is very low, then predators might not engage at all in searching and in this way save the energy cost of movement.

Future studies should follow not only the vipers but also document the vegetation surrounding their track to examine whether the movement is more tortuous in areas with dense vegetation. Another direction should be to examine whether the viper activity or decision which foraging mode to employ is correlated with prey abundance. For example, larger gerbil species are active earlier in the night than smaller ones, which overlaps with the viper's activity time [69]. One could expect active foraging to reach its peak in medium levels of prey while both low and high abundance should lead to lower activity [41,70]. Foraging mode should also depend on prey mobility [41,71] so uncovering what the common prey in each season is should be valuable. Related gerbil species partition activity time during the night [69]. It is an interesting and open question whether sand vipers do so too, as the larger *C. cerastes* co-occurs with the smaller *C. vipera* in the same habitats [34]. Furthermore, although the vipers use both ambush and active foraging, the allocated time to each of the two foraging modes is unknown. Finally, potential considerations of predation risk by predators of the viper and thermoregulation are important to examine.

## 5. Conclusion

Our study provides important information on the behaviour and ecology of *C. cerastes*, such as movement patterns, activity time and inter-sexual differences. The next step should be to design experiments to test the patterns discovered here. Only by targeted manipulations of single factors, such as prey abundance or habitat structure, one can truly understand what the cause and effect are, and the triggers of behavioural flexibility in the studied viper. As sand dunes in Israel, mainly the coastal ones but also those in the desert, are threatened owing to multiple reasons, it is important to examine the behaviour, habitat preference and population size of animals occurring in such habitats. Such studies may later help in designing conservation plans.

## Data Availability

The datasets supporting this article is uploaded as part of the electronic supplementary material [72].
